# Seasonality of hospital admissions for mental disorders in Hanoi, Vietnam

**DOI:** 10.3402/gha.v9.32116

**Published:** 2016-08-25

**Authors:** Phan Minh Trang, Joacim Rocklöv, Kim Bao Giang, Maria Nilsson

**Affiliations:** 1 Department of Public Health and Clinical Medicine, Epidemiology and Global Health, Umeå University, Umeå, Sweden; 2 Institute for Preventive Medicine and Public Health, Hanoi Medical University, Hanoi, Vietnam

**Keywords:** seasonality, summer, temperature, mental disorders, daily hospital admissions

## Abstract

**Background:**

Some studies have shown a relationship between seasonality in weather patterns and depressive and behavioural disorders, especially in temperate climate regions. However, there is a lack of studies describing the seasonal patterns of hospital admissions for a variety of mental disorders in tropical and subtropical nations. The aim of this study has been to examine the relationship between seasons and daily hospital admissions for mental disorders in Hanoi, Vietnam.

**Designs:**

A 5-year database (2008–2012) compiled by Hanoi Mental Hospital covering mental disorder admissions diagnosed by the International Classification of Diseases 10 was analysed. A negative binominal regression model was applied to estimate the associations between seasonality and daily hospital admissions for mental disorders, for all causes and for specific diagnoses.

**Results:**

The summer season indicated the highest relative risk (RR=1.24, confidence interval (CI)=1.1–1.39) of hospital admission for mental disorders, with a peak in these cases in June (RR=1.46, CI=1.19–1.7). Compared to other demographic groups, males and the elderly (aged over 60 years) were more sensitive to seasonal risk changes. In the summer season, the RR of hospital visits among men increased by 26% (RR=1.26, CI=1.12–1.41) and among the elderly by 23% (RR=1.23, CI=1.03–1.48). Furthermore, when temperatures including minimum, mean, and maximum increased 1°C, the number of cases for mental disorders increased by 1.7%, 2%, and 2.1%, respectively.

**Conclusion:**

The study results showed a correlation between hospital admission for mental disorders and season.

## Introduction

The World Health Organization (WHO) has recognised mental and neurological disorders, including Alzheimer's disease, as important diseases contributing essentially to the global non-communicable burden of disease and disability (1). In 2004, mental disorders accounted for 13% of the global disease burden. The proportion of patients living with disability from mental disorders accounted for 25.3 and 33.5% in low- and middle-income populations, respectively (1). More than 450 million people suffer from mental disorders, and among those depression, dementia, epilepsy, and schizophrenia are amounting to 350, 35.6, 50, and 24 million people, respectively. According to WHO, major depressive disorder may become the principal cause of disability worldwide by 2030 (2). The global burden of disease study from 2010 found that mental, neurological, and substance consumption disorders contributed to 10.4% of global disability-adjusted life years (DALYs) as well as 28.5% of global years lived with disability (YLDs) (3). Mental disorders accounted for the highest proportion of DALYs (56.7%) compared with neurological disorders (28.6%) and substance use disorders (14.7%) (3). Moreover, mental and substance consumption disorders became the leading cause of YLDs all over the world. There were 40.5, 14.6, 10.9, 9.6, and 7.4% of DALYs for depressive disorders, anxiety disorders, drug use disorders, alcohol abuse, and schizophrenia, respectively (3, 4).

A study carried out in 17 nations on lifetime prevalence of mental disorders showed that there were risks among Asian populations, ranging from 13.2% in China and 14.4% in Iran to 18.0% in Japan (5). No official studies at the national level on mental disorders have been conducted in Vietnam. However, a representative epidemiological survey in Vietnam covering 10 common mental disorders in the population between 2001 and 2003 indicated a prevalence of 14.9%, of which alcohol abuse, depression, and anxiety accounted for 5.3%, 2.8%, and 2.6%, respectively (6).

Many studies have described a relationship between seasonality and depressive disorders, particularly in countries with temperate climate characteristics (7–9). Most of the previous research on depressive disorders and seasonality has shown evidence of a spring/summer peak incidence for manic episodes (10, 11). For example, hospital entries for manic episodes in England revealed a summer peak. Research in Australia and New Zealand also indicated a similar peak, with the highest rate for manic episodes in the warm spring/summer season (from December to February) (10, 12–14).

Several studies have identified more cases of patients with schizophrenia in the summer. Mechanistically, high ambient temperature has been suggested to contribute to more frequent onset of psychotic exacerbation (15). Research in the Northern Hemisphere including Ireland, England, and Scotland has shown a seasonal influence on first admissions for schizophrenia, and the peak time of the first hospital entries for mental disorders occurs in the middle of the year (15, 16). Research that was conducted in Israel showed that there was a significant relationship between higher ward temperature and more severe symptoms among inpatients with schizophrenia (16).

Ambient temperature has long been suspected of being a cause of psychotic exacerbation in mental and behavioural disorders (17). Several studies from both developed and developing nations have indicated an increase in hospital admissions and emergency room visits during periods of a rise in temperature for individuals with psychological problems (9, 16, 18, 19).

As we can see, there is evidence of a relationship between specific mental disorders, seasonality, and temperature variation with an increased trend of hospital admissions for mental disorders. However, most research was conducted in developed populations where weather characteristics, socio-economic conditions, and culture differ from those in Vietnam. It is therefore important to examine the association between variations in mental disease admission and season in Hanoi, Vietnam.

## The study aim

The aim of this study has been to examine the relationship between seasons, mean temperature, and daily hospital admissions for mental disorders in Hanoi, Vietnam.

## Methods

### Hospitalisation data

One of the two big mental hospitals taking care of mental patients, Hanoi Mental Hospital has captured both emergency visits and outpatients from Hanoi City and neighbouring provinces. In this study, we analysed a database from the hospital that covered mainly emergency cases for mental disorders in Hanoi City.

All hospital admissions for mental diseases in Hanoi, in northern Vietnam, were collected from Hanoi Mental Hospital over a 5-year period from 2008 to 2012. The hospital admissions were diagnosed by mental health specialists from the hospital using the International Classification of Diseases 10 (ICD 10). Each patient was assigned a principal diagnostic ICD-10 code. ICD 10 classifies mental disorders as codes F00–F99. In this study, mental disorders were classified into eight groups of psychiatric disorders comprising Organic, including symptomatic, mental disorders (F0–9); Mental and behavioural disorders due to psychoactive substance use (F10–19); Schizophrenia, schizotypal, and delusional disorders (F20–29); Mood (affective) disorders (F30–39); Neurotic, stress-related, and somatoform disorders (F40–48); Behavioural syndromes associated with physiological disturbances and physical factors (F50–59); Mental retardation (F70–79); and Unspecified mental disorder (F99).

### Meteorological data

Located at 21°2′0′′N 105°51′00′′E, Hanoi has a typical subtropical weather with four defined seasons. Hanoi's area of 3,329 km^2^ includes 13 districts (referred to as the urban area) and 17 suburbs (referred to as the rural area). Hanoi, the capital of Vietnam, has a population of around 7 million people living in a typical subtropical climate with four seasons. Seasons are defined as follows: spring (March–May), summer (June–August), autumn (September–November), and winter (December–February). In recent decades, the mean daily temperatures have steadily increased, affected by climate change and urbanisation (20).

Meteorological data including daily maximum, minimum, and mean temperatures were collected from several monitoring stations in the Hanoi region, including the same urban and rural areas for the period 2008–2012. Data from the different monitoring stations were summed to estimate an average of a city mean value. The daily mean values of ambient temperature were used to estimate a daily increase in mental disorder admissions associated during the 5 years.

**Figure:**
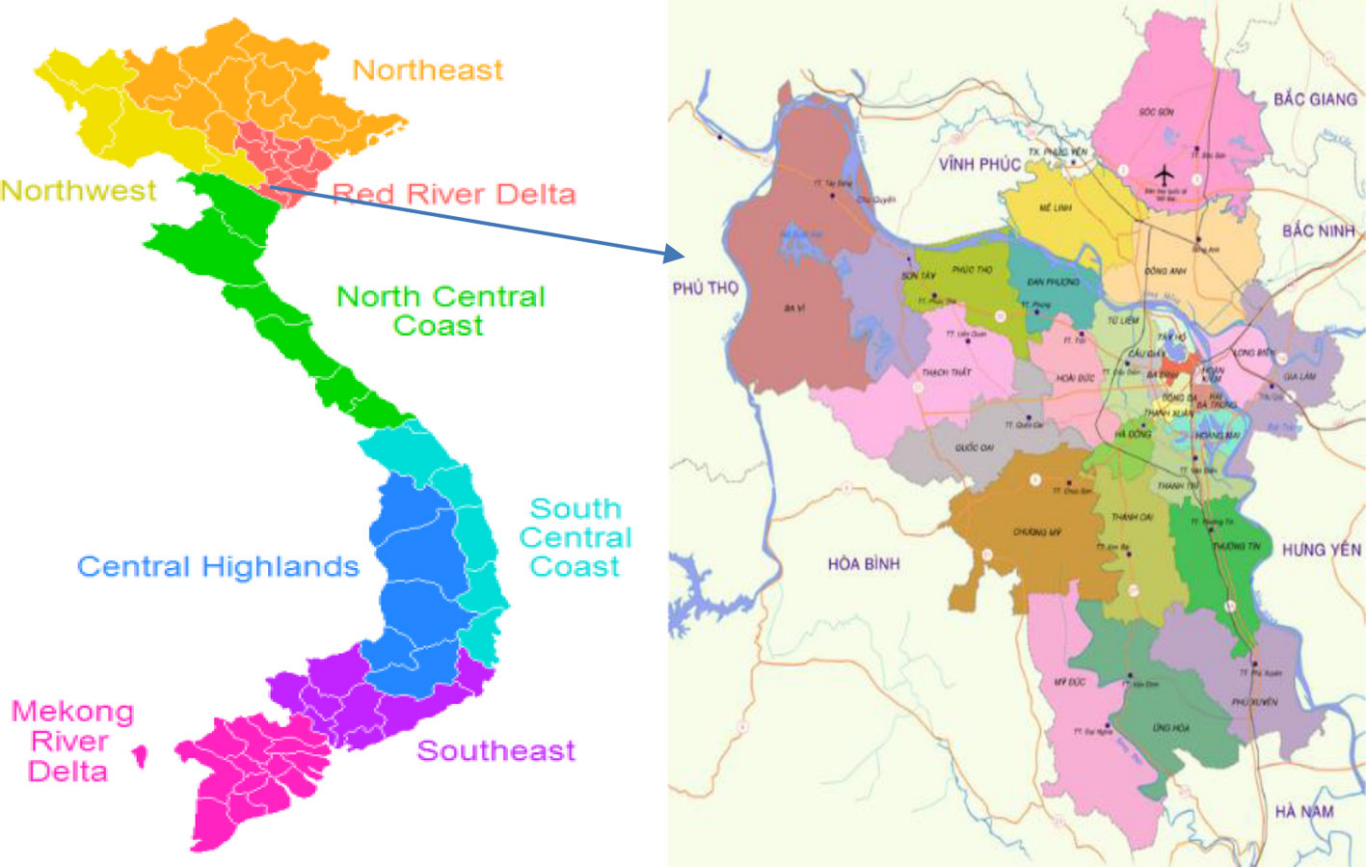
A map of Hanoi, Vietnam

### Statistical analysis

A negative binomial regression model for time series was applied to estimate the relationship between daily hospital admissions as a function of season and month, for which the winter season and January were used as annual reference times. We adjusted for year in the analysis to remove confounding of time trends.

Moreover, we also established negative binomial time-series regression models of the aggregated counts of daily admissions as an outcome variable and indicator variables for daily temperature variations, the day of the week, season, and the time trend in the study period as explanatory variables.

An electronic data set with 5 years (2008–2012) from Hanoi Mental Hospital including identified code, the date and time of admission, the age for both children and adults, gender, diagnosis of mental disorders, and the treatment for mental disorders were anonymised and identified prior to analysis. In addition, the daily hospital admissions for psychiatric disorders were analysed by stratifying by age, sex, and geographic location. Estimates were generated as relative risks (RRs) with 95% confidence intervals.

### Ethics statement

Electronic health data sets including the code, age, sex, date, and treatment of admissions for mental disorders, both children and adults, were anonymised and de-identified prior to analysis. All procedures were approved by Hanoi Medical University and the Mental Hospital Ethics Board in Vietnam.

## Results

### Weather variation

**Fig. 1:**
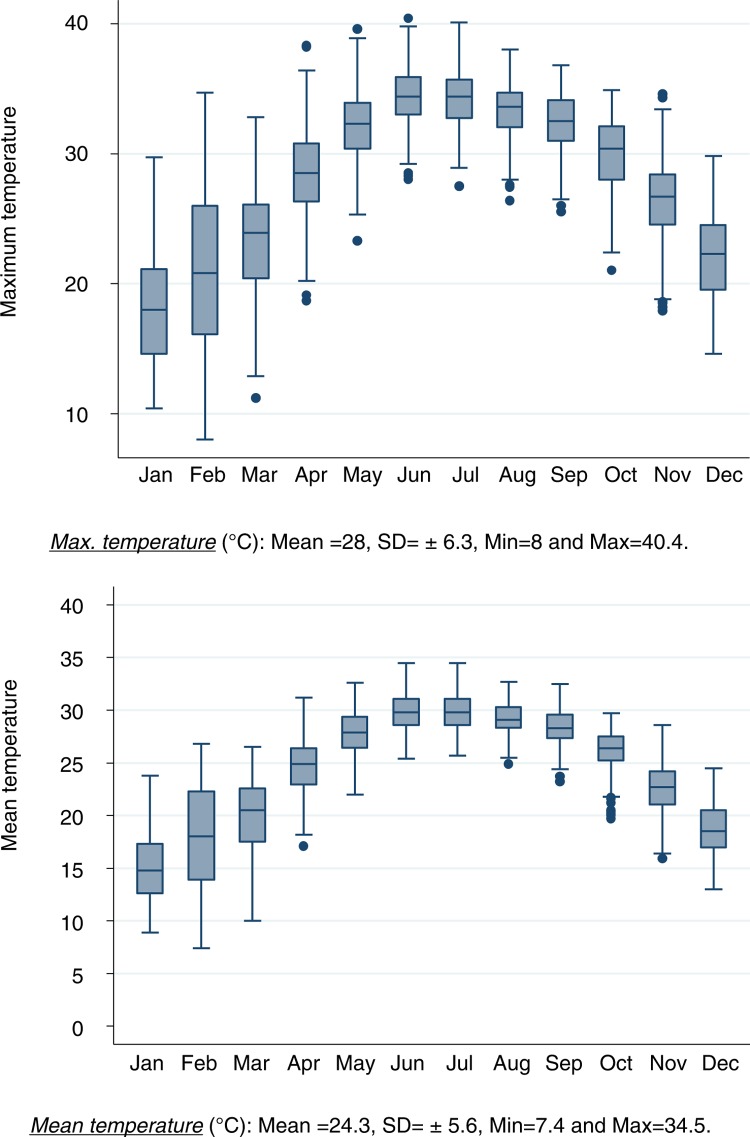
The average of monthly mean temperature and maximum temperature during the 5-year period 2008–2012. *Max. temperature* (°C): Mean =28, SD=± 6.3, Min=8 and Max=40.4; *Mean temperature* (°C): Mean=24.3, SD=± 5.6, Min =7.4 and Max=34.5.

During the 5-year period (2008–2012), the seasonal temperature reached a high of around 30°C in summer (June–August) and a low of 15–17°C in winter (December–February). The average daily mean temperature was 24.3°C with a standard deviation of±5.6°C. In addition, the average of the daily maximum temperatures was 28°C, with a daily maximum of 40.4°C. The daily maximum temperatures in the winter and summer seasons were around 18–21°C and 33°C, respectively. In Hanoi, during the same study period, the month of June had the hottest weather with an average of daily maximum and mean temperatures of around 30°C±2°C and 34.4°C±2.4°C (standard deviations), respectively (Fig. 1).

### Hospital admissions for mental disorders

**Fig. 2:**
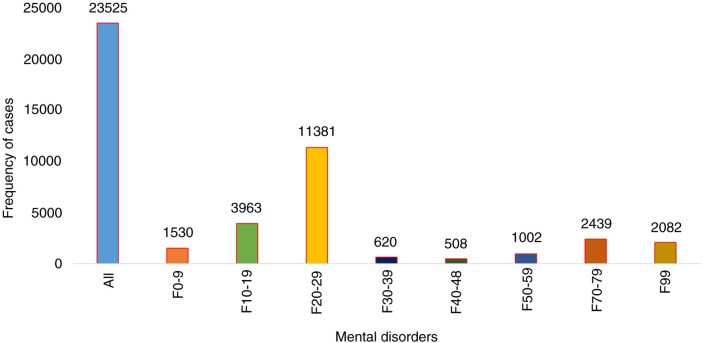
The number of cases for specific mental disorders during 5 years 2008–2012**. F0–9: Organic, including symptomatic, mental disorders; F10–19: Mental and behavioural disorders due to psychoactive substance use; F20–29: Schizophrenia, schizotypal, and delusional disorders; F30–39: Mood (affective) disorders; F40–48: Neurotic, stress-related, and somatoform disorders; F50–59: Behavioural syndromes associated with physiological disturbances and physical factors; F70–79: Mental retardation; F99: Unspecified mental disorder.

**Fig. 3:**
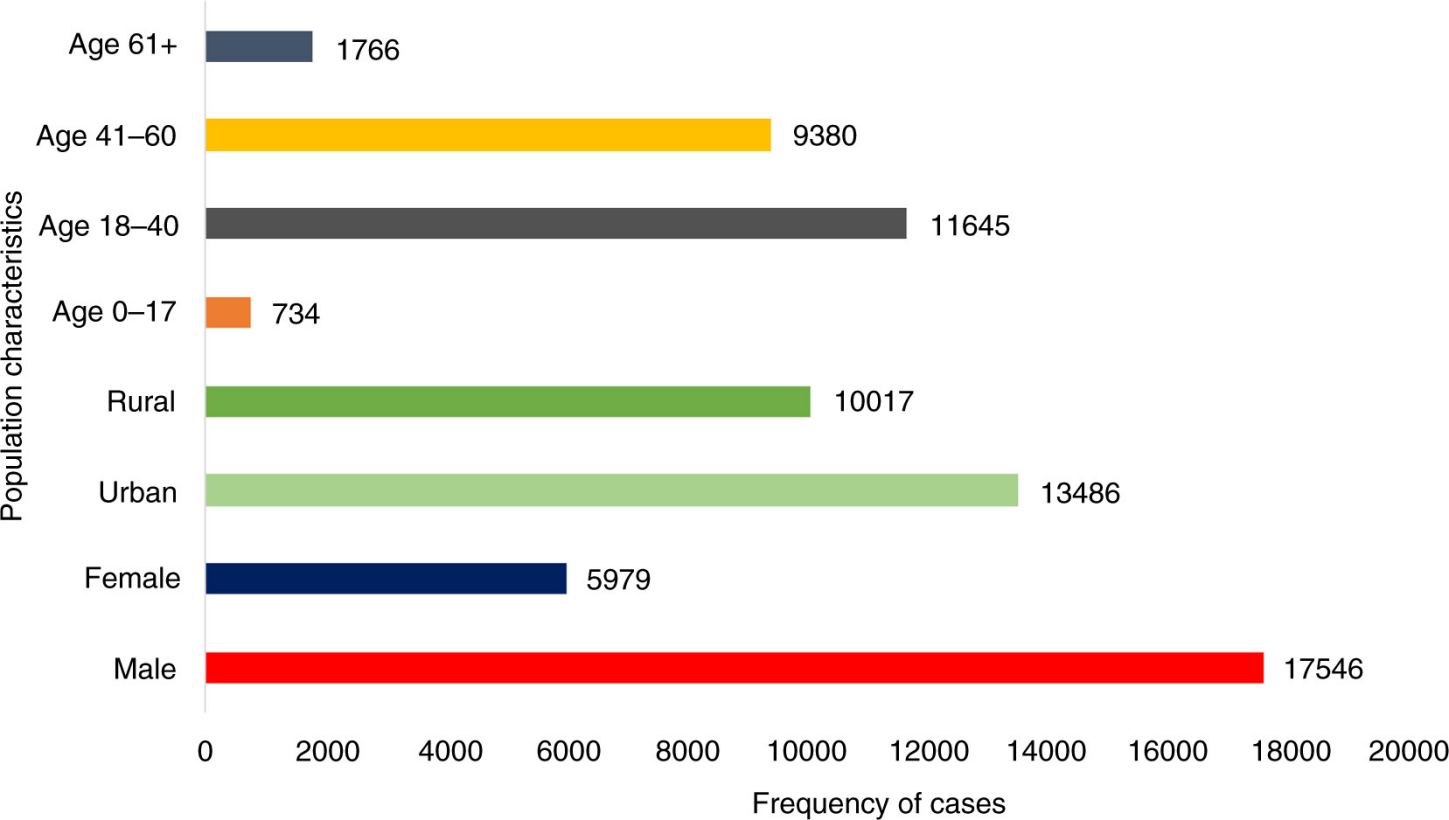
The characteristics of study population.

Throughout the study period (2008–2012), 23,525 subjects were admitted to the hospital with psychiatric problems, including both inpatients and outpatients (this refers to the first time they were registered at Hanoi Mental Hospital; see Fig. 2). During this period, the numbers of male and female patients with mental disorder admissions were 17,546 (74.6%) and 5,979 (25.4%), respectively; the numbers of admissions of patients from urban and rural areas were 13,486 (57.3%) and 10,017 (42.6%), respectively, whereas the age distribution in groups 0–17, 18–40, 41–60, and over 60 years was 734 (3.1%), 11,645 (49.5%), 9,380 (39.9%), and 1,766 (7.5%), respectively (Fig. 3).

### The relationship between seasonality and mental disorders

**Fig. 4:**
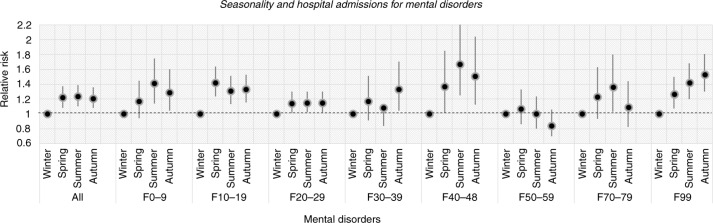
The relationship between daily hospital admissions for mental disorders and seasonality. F0–9: Organic, including symptomatic, mental disorders; F10–19: Mental and behavioural disorders due to psychoactive substance use; F20–29: Schizophrenia, schizotypal, and delusional disorders; F30–39: Mood (affective) disorders; F40–48: Neurotic, stress-related, and somatoform disorders; F50–59: Behavioural syndromes associated with physiological disturbances and physical factors; F70–79: Mental retardation; F99: Unspecified mental disorder.

**Fig. 5:**
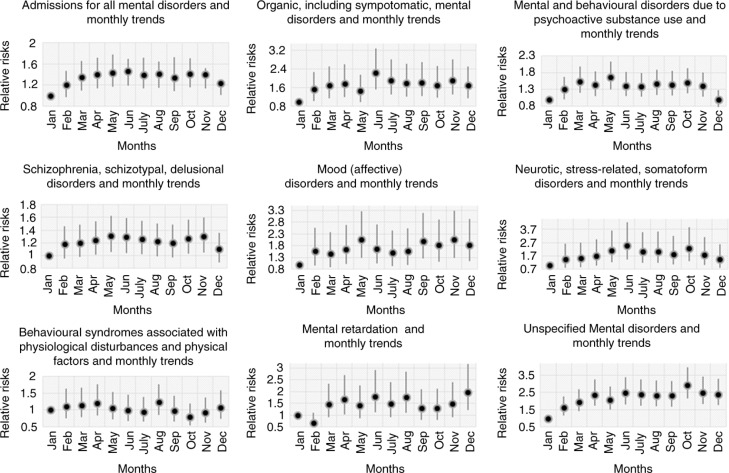
The relation between daily hospital admissions for mental disorders and monthly trends.

*For Mental and behavioural disorders* (ICD-10 codes F00–99), during the study period, the number of cases of mental disorders had a seasonal high in the summer season (RR=1.24, CI=1.1–1.39) with a peak in June (RR=1.46, CI=1.19–1.7; Figs. 4 and 5). The annual seasonal low occurred in the winter season (the reference season). The RRs of the mental disorders estimated an increase in the autumn season (RR=1.20, CI=1.08–1.36) and the spring season (RR=1.22, CI=1.08–1.37; Fig. 4). In general, all groups of hospital admissions for mental disorders showed higher risks of admission during the hotter months from May to August (Fig. 5).

*For Organic, including symptomatic, mental disorders* (ICD-10 codes F00–09), the number of patients admitted estimated to 1,530 (6.5%), with higher rates in the summer season (RR=1.41, CI=1.14–1.75) than in the spring season (RR=1.17, CI=0.94–1.45) and autumn season (RR=1.29, CI=1.04–1.6; Figs. 3 and 4). The annual peak of cases occurred in June (RR=2.24, CI=1.53–3.29; Fig. 5).

*For Mental and behavioural disorders due to psychoactive substance use* (ICD-10 codes F10–19), the number of cases coming under F10–19 accounted for 3,963 (16.8%) admissions (Fig. 3). Hospital visits peaked in the spring season (RR=1.42, CI=1.23–1.64) with slightly lower risks in summer (RR=1.31, CI=1.13–1.51) and autumn (RR=1.33, CI=1.15–1.53; Fig. 4). The greatest risk increase was seen in May (RR=1.66, CI=1.3–2.13; Fig. 5).

*For Schizophrenia, schizotypal, and delusional disorders* (ICD-10 codes F20–29), the highest proportion of cases among all diagnoses occurred and amounted to 11,381 (48.4%) patients (Fig. 3). However, this group had a less strong seasonal difference in admissions over the year. Nevertheless, admissions increased in the summer (RR=1.15, CI=1.02–1.3), autumn (RR=1.15, CI=1.02–1.3), and spring (RR=1.15, CI=1.01–1.29) compared to admissions in the winter (Fig. 4). The peak of cases was seen in May and June with RRs of 1.31 (CI=1.06–1.62) and 1.29 (CI=1.04–1.59) respectively (Fig. 5).

*For Mood (affective) disorders* (ICD-10 codes F30–39), the number of 620 hospital visits peaked in the autumn (RR=1.33, CI=1.04–1.7) and was slightly higher in the summer (RR=1.08, CI=0.84–1.4) and spring (RR=1.17, CI=0.91–1.51) than in the winter (Fig. 4). Admissions peaked sharply in May and November with RRs of 2.05 (CI=1.29–3.26) and 2.05 (CI=1.29–3.27), respectively (Fig. 5).

*Neurotic, stress-related, and somatoform disorders* (ICD-10 codes F40–48) include anxiety disorders, panic disorder, agoraphobia, obsessive compulsive disorder, and posttraumatic stress disorder. The total number of hospitalisations was 508 (2.2%) during the study period, with a peak in the summer season (RR=1.67, CI=1.25–2.25) and with slightly lower increases in the autumn season (RR=1.51, CI=1.12–2.04) and spring season (RR=1.37, CI=1.01–1.85; Fig. 4). From May to August, the number of admissions posed the highest risk with RRs from 2.11 to 2.47, especially in June (RR=2.47, CI=1.45–4.22; Fig. 5).

*For Behavioural syndromes associated with physiological disturbances and physical factors* (ICD-10 codes F50–59), cases peaked in the spring season (RR=1.07, CI=0.86–1.33) and in August (RR=1.23, CI=0.85–1.77) (see Fig. 5). This category involves eating and sleeping disorders and amounted to 1,002 (4.3%) admissions (Fig. 3).

*For Mental retardation* (ICD-10 codes F70–79), admissions encompassing mild, moderate, and severe as well as profound intellectual disabilities accounted for 2,439 (10.4%) cases (Fig. 3). There were differences among three of the seasons, where the number of cases in the summer (RR=1.36, CI=1.03–1.8) was higher than that in the autumn (RR=1.09, CI=0.82–1.44) and spring (RR=1.23, CI=0.93–1.63) relative to that in the winter season (Fig. 4). Admissions peaked in June and August with RRs of 1.79 (CI=1.11–2.9) and 1.76 (CI=1.1–2.83), respectively (Fig. 5).

*For Unspecified mental disorder* (ICD-10 codes F99), there were estimated to be 2,082 (8.8%) admissions in this category (Fig. 3). The highest RR for admissions was found in the autumn season (RR=1.53, CI=1.3–1.8), whereas the RRs in the summer and spring seasons were 1.42 (CI=1.2–1.68) and 1.27 (CI=1.07–1.5), respectively (Fig. 4). The peak of admissions for unspecific mental disorders reached an annual high in October (RR=2.92, CI=2.14–3.97; Fig. 5).

### The relationship between seasonality and mental disorders stratified by sex, location, and age

**Fig. 6:**
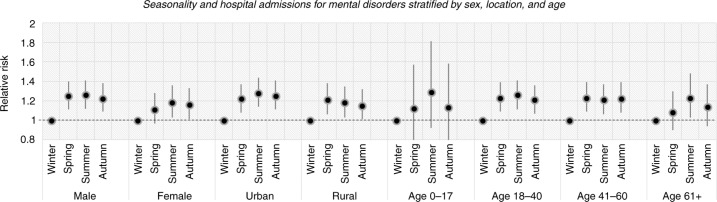
The association between daily hospital admissions for mental disorders and seasonality stratified by sex, location, and age.

**Fig. 7:**
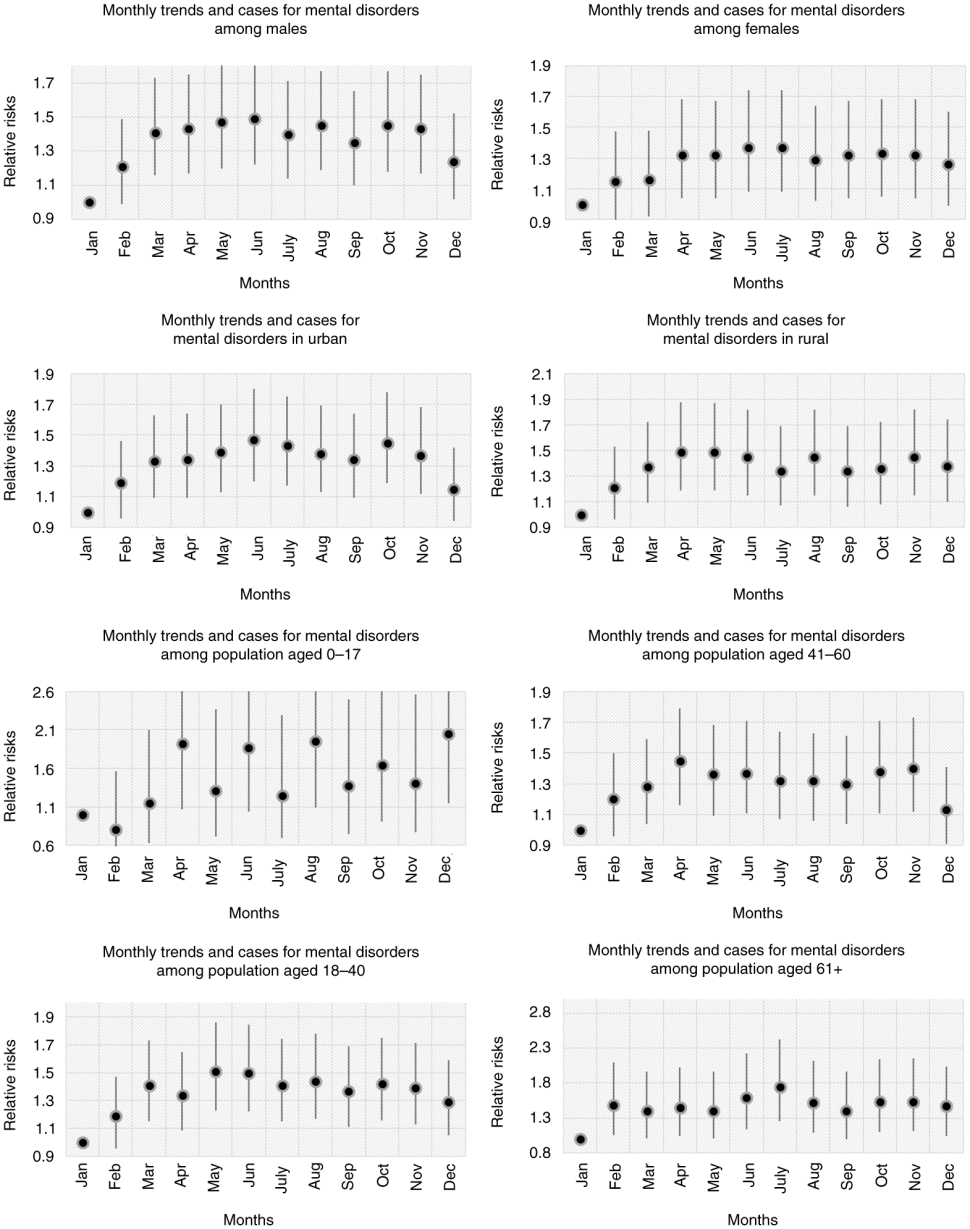
The associations between daily hospital admissions for mental disorders stratified by sex, location and age groups.

In the summer season, hospital admissions for mental disorders among males (RR=1.26, CI=1.12–1.41) were more common than for those among females (RR=1.18, CI=1.03–1.36) compared to the winter season. A peak in cases in both men and women was observed in early summer with RRs of 1.49 (CI=1.22–1.82) among men and 1.37 (CI=1.08–1.74) among women (Figs. 6 and 7) compared to January.

Admissions among residents in urban and rural areas increased in the summer season (RR=1.28, CI=1.14–1.44) and spring season (RR=1.21, CI=1.06–1.38) compared to the winter season (Fig. 6). Moreover, the peaks in cases in urban and rural areas occurred in June with an RR of 1.47 (CI=1.2–1.8) and in May with an RR of 1.49 (CI=1.19–1.87) respectively compared to January (Fig. 7).

In this study, those aged 0–17 years accounted for the highest risk of mental disorders in the summertime (RR=1.29, CI=0.92–1.81) (Fig. 6). A peak in cases was seen in August with an RR of 1.95 (CI=1.09–3.47). Among the elderly, the number of admissions for mental disorders was also high in the summer season and especially in July with RRs of 1.23 (CI=1.03–1.48) and 1.75 (CI=1.26–2.42), respectively. In addition, the group aged 18–40 years had the highest risk of mental disorders in the summer (RR=1.26, CI=1.11–1.41) and in May (RR=1.51, CI=1.23–1.86). The highest rates among patients aged 41–60 years (RR= 1.23, CI= 1.09–1.39) were reported in the spring season (RR= 1.45, CI= 1.16–1.7), especially in April (Fig. 7).

### The relationship between temperatures and mental disorders

In this study, when minimum, mean, and maximum temperatures increased 1°C, the number of cases for mental disorders increased by 1.7%, 2%, and 2.1%, respectively (Table 1).

**Table 1:** The relationship between temperatures and daily hospital admissions for mental disorders during 5 years (2008–2012)

Admissions	Relative risk	Confidence interval (95%)
Max. temperature	1.017	1.004–1.03
Mean temperature	1.021	1.005–1.04
Min. temperature	1.020	1.003–1.04

**Fig. 8:**
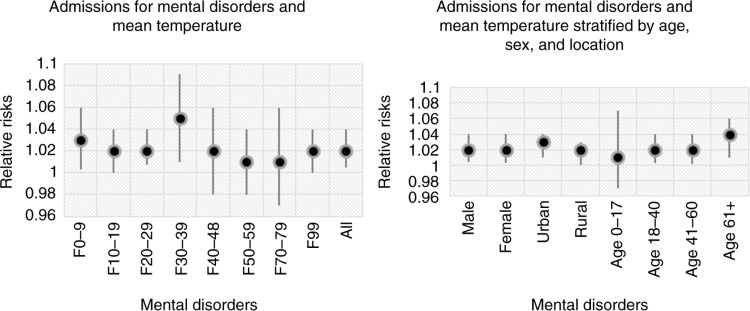
The relationship between daily hospital admissions for mental disorders and the variation in ambient mean temperature.

Among patients with mood disorders (F30–39), it increased 5% (RR =1.05, CI=1.01–1.09; Fig. 8). The group of the elderly had the highest risk of mental disorders when mean temperature increased one unit (1°C) with RR of 1.04 (1.01–1.06) (Fig. 8).

## Discussion

We found a correlation between hospital admissions for mental disorders and season in which the number of hospital visits for mental disorders increased by 24% in the summer season with a peak in these admissions in June. Compared to other demographic groups, males and the elderly (aged over 60 years) were more sensitive to seasonal risk changes. The RR of hospital admissions among men increased by 26% and among the elderly by 23% in the summer season.

The study results show that the admission rates for males were more than three times higher than those for females. According to the literature in mental health, admission rates for psychiatric problems among males are always higher than those among females, except in the case of depression disorders (21). In this study, patients with mental disorders living in rural regions were similar to those living in urban regions. There were differences in hospital entries in between age groups whereby the group aged 18–40 years accounted for the highest rate. Moreover, the greatest proportion of mental disorders accounted for the number of cases of schizophrenia (37.17%), in contrast to a WHO report which states that the rate of depressive disorders is usually higher than that of schizophrenia (1, 2).

In this study, mood disorders (F30–39) and unspecified mental disorders (F99) had the highest risks in the autumn season. The results were similar to the findings in research on depressive disorders and seasonality, especially in countries with a temperate climate (22, 23). However, when taking into account monthly trends in mental disorders, admissions of mood disorders (F30–39) and unspecified mental disorders (F99) showed quite high risks in May and June. Studies in Brazil, India, and Egypt showed a high number of cases of mood disorders in the hotter months (15, 24).

The results of this study indicated, however, that there was a peak in admissions for mental disorders in the summer season with its fairly extreme weather pattern of high temperatures. The groups of mental disorders including psychoactive substance use (F0–9), schizophrenia (F20–29), somatoform disorders (F40–48), and mental retardation (F70–79) had the highest risks during the summer season. Moreover, our data demonstrated positive associations between temperature and admissions for mental disorders both for all and for specific mental disorders in the month of June when the weather is at its hottest. This increase in summertime may explain the increase in the number of cases of mental disorders and vice versa. Research worldwide has studied the relationship between ambient temperature and schizophrenia or depressive disorders, whereas other specific mental diseases such as dementia and drug and substance abuse have not been studied enough (19, 15, 25, 26). A relationship was reported between environmental temperature and schizophrenia morbidity, where the mean maximum monthly environmental temperature was associated with the monthly intake of inpatients suffering from schizophrenia especially during the summer season (16, 25). Similar findings have been mentioned in other research conducted in both developed and developing countries (15, 19, 27, 28). Lane K et al. reported from the United States that there was a statistically significant difference in the relationship between ambient temperature and visits to a psychiatric emergency department for psychiatric problems (27). In addition, Hansen et al. in Australia indicated that admissions during 1993–2006 for behavioural and mental disorders were correlated with a maximum daily temperature above a threshold of 26.7°C (18). Similar outcomes have been found in other countries such as France, Spain, India, and Israel, whereby France had a high rate of visits to a psychiatric emergency department during the period of heatwaves in 2003 (25, 29–31). Several studies conducted in Israel showed that there was a higher risk of admissions for schizophrenia when this was associated with mean maximal monthly temperature (16, 25). Similarly, scientists in India reported that there was a greater use of psychiatric services by patients with mood disorders in the summer when temperatures were at their hottest (9). The impact of daily climatic variables on psychosis admissions to mental hospitals as reported by the Irish Health Research Board revealed that a weak association existed between temperature and psychosis cases (28). Furthermore, there were found to be acute effects of extreme temperature exposure on emergency room admissions for mental and behavioural disorders in Toronto, Canada, where there was a strong relationship between these at 28°C, especially within a period of 0–4 days of exposure to hot weather. An estimate of cases showed that there was an increase of 29% over a cumulative period of 7 days after exposure to mean high ambient temperature in the 99th compared with the 50th (19).

In our study, there was a significant difference in the gender distribution, in which the number of hospital admissions for males was greater than for females, especially in hot weather summer and spring seasons. Hospital visits for mental disorders among men were threefold compared to those among women. Previous studies of mental diseases indicated that the proportion of male patients with mental disorders was larger than the proportion of female patients, except for depressive disorders (21). In Vietnam, there have been high proportions of alcohol consumption and brain damage due to traffic accidents among men. Thus, this may contribute to the increased risk of mental disorders for male patients, especially for the groups with alcohol abuse, and delirium (32, 33). However, further studies need to be conducted in the future to ascertain the gender difference in the association between mental disorders and heat/heatwave exposure.

Our data also confirmed that there were higher rates of cases in rural populations in May and June. This result corresponded to findings from Australia and Taiwan because an increased impact of hot weather on rural mental health had been observed (34, 35). It is known that high vulnerability in rural communities can be predicted during variations or extremes in weather (36). In addition, the number of cases of mental disorders among residents in urban regions also increased in the summertime, with a peak in June. This may be explained by a rise in temperatures and the problems of urbanisation (20). Regarding the association between admissions and seasonality and month trends between age groups, the group aged 0–17 years had peaks of mental disorders, especially in summertime and August. There were increased trends in daily hospital admissions for psychiatric disorders among other age groups such as those aged 18–40 and 41–60 years, where the number of cases rose in hot weather including the spring and summer seasons and especially in April, May, and June. In this study, patients over 60 years of age peaked in the summer period and July. The elderly had a higher risk of mental disorders than did others on the basis of monthly trends from February to December, and especially in June and July.

When the average of temperatures including minimum, mean, and maximum increased one unit (1°C), the number of cases for mental disorders rose between 1.7 and 2.1% in which patients with depressive disorders accounted for the highest risk with an increase by 5%. Findings from Korea, Taiwan, Australia, Brazil, and Egypt indicated that admissions for depressive disorders increased with rising ambient temperature (18, 15, 24, 26, 34). Thus, emerging admissions beyond elevated temperature may explain the peak of daily hospital visits in hottest periods including June and July. Furthermore, when mean temperature increased one unit (1°C), the number of cases for mental disorders in the population aged over 60 years rose by 4%. The results were similar to the outcome of a study conducted in Australia on heat waves and mental disorders, in which the elderly population (65–74 years of age) had the highest risk of suffering mental problems (18).

Psychological responses including thermal sensation and human behaviour are greatly affected by thermal environment (37). In spite of the prime importance attached to them, psychological studies on the impact of thermal environment are still at their infancy (18, 38). According to Hensel (1981), thermo-reception resulting in qualities such as warmth is qualitative, originating as it does from sensory experience, and it therefore cannot be based on physics or physiology (37). In addition, another approach taken by research has been to study the association between environmental conditions, physiological responses, and psychological phenomena such as behaviour and sensation (11, 18, 37). Elevated temperature can exacerbate mental and behavioural disorders for a variety of physiological and psychological reasons in which vulnerable neurotransmitters and thermoregulation play in part a key role (36, 37, 39, 40). This has been demonstrated in animal models of impaired dopaminergic transmission in schizophrenia (19, 21). Moreover, many findings on homeostasis maintain that while the body was in the state with mental disorders, its neurotransmitters, including serotonin, dopamine, and norepinephrine, underwent change when environmental change was influenced by ambient temperature (40, 41). Several experiments in mice and rats have proven the impact of ambient temperature on drug-induced dopamine and serotonin neurotoxicity (42). Some medications affecting neurotransmitters may influence thermoregulation, and some can trigger malignant hyperthermia, neuroleptic syndrome, or serotonin syndrome (21, 43). Physiological mechanism-related factors associated with high temperature, brain temperature, and mental-psychological disorders may shed light on the hypothesis in the study of whether the summer season with its high temperatures may aggregate hospital admissions for mental disorders. However, further research on biological, psychological, and physiological areas is required to find out the exact mechanisms associated with heat-related mental illnesses.

Climate change has become a big challenge worldwide (44). The effects of rising temperatures on human health, both physical and mental, come through direct and indirect pathways (36, 45). There has been a lack of studies on mental health consequences, but recent studies have shown a high risk of heat-related mortality and morbidity with an increasingly heat-associated mental illness (18, 19, 45–47). Vietnam situated in the subtropics is suffering annual droughts and heat waves. It is one of the nations with a high potential of being most negatively impacted by climate change with temperatures rising by 0.5–0.7°C per 50 years, from the South to the North (48). Moreover, the mean temperatures in all regions in Vietnam are projected to increase by 1.3–1.6°C, 1.6–1.9°C, and 1.9–2.2°C in 2050, 2070, and 2100, respectively, compared with the period 1980–1999 (48). The trend of increased temperatures in Vietnam may most likely increase the risk for heat-related mortality and morbidity in the future, both physical and mental. This study illustrated that the peak of admissions for mental disorders was associated with the warmest season in Vietnam. Thus, further studies on the impact of extreme heat and heat waves on mental disorders and vulnerable populations in Vietnam are very important and considered necessary to conduct.

## Conclusion

There was an association between hospital admissions for mental disorders and seasonality with peaks of cases occurring in the hot weather of the summer season, especially in June. There were gender and age group differences where a significantly higher number of men and elderly patients (>60 years of age) were admitted for mental disorders in times of hot weather. In the planning of mental health care, the results can be used as an indication of expected peaks of mental ill health in relation to seasons and temperatures, and as a guide for resource allocation. It also gives clinical doctors of mental health in Vietnam more information regarding mental diseases and their associations with seasons and hot weather. By having this understanding they can be better prepared. The study results are formative and may pave the way for future research in the region. Further studies will be needed in the future to explore the impacts of prolonged heat waves on mental-psychological disorders, especially in populations at risk. It may help health managers to be better prepared, by setting up good strategies to prevent heat-related mental illnesses and protect vulnerable populations, at risk of mental health problems.

## Limitations of the study

The 5-year database from one psychiatric hospital in northern Vietnam was used to estimate the relationship between seasonality and admissions of mental cases. This may not capture fully all mental disorder patients in Hanoi, especially outpatients. Moreover, there are different regions in Vietnam which have distinctly specific weather patterns; therefore, it is impossible to generalise the results to the national level. This is a formative study. More studies are needed in the future using more information to build a model by taking into account the environment, personal traits, and socio-economics together with mental health problems in order to identify vulnerable populations.

## Paper context

Several studies on seasonality and mood disorders, and schizophrenia have been conducted worldwide, especially in the developed countries. Vietnam, a low-and middle-income nation, has increasingly suffered consequences of extreme heat/heatwaves. Thus, research studies on relationships between mental disorders and seasonality, heat exposure, as well as on limitations of its consequences, are very important. It is difficult to avoid natural disasters, but we can prevent heat-related mental illnesses, particularly for poor people who need real help and protection.
